# Filament Condition-Specific Response Elements Control the Expression of *NRG1* and *UME6*, Key Transcriptional Regulators of Morphology and Virulence in *Candida albicans*

**DOI:** 10.1371/journal.pone.0122775

**Published:** 2015-03-26

**Authors:** Delma S. Childers, David Kadosh

**Affiliations:** Department of Microbiology and Immunology, University of Texas Health Science Center at San Antonio, San Antonio, United States of America; Louisiana State University, UNITED STATES

## Abstract

*Candida albicans* is the most frequently isolated human fungal pathogen and can cause a range of mucosal and systemic infections in immunocompromised individuals. Morphogenesis, the ability to undergo a reversible transition from budding yeast to elongated filaments, is an essential virulence trait. The yeast-to-filament transition is associated with expression of genes specifically important for filamentation as well as other virulence-related processes, and is controlled, in part, by the key transcriptional regulators Nrg1 and Ume6. Both of these regulators are themselves controlled at the transcriptional level by filament-inducing environmental cues, although little is known about how this process occurs. In order to address this question and determine whether environmental signals regulate transcription of *UME6* and *NRG1* via distinct and/or common promoter elements, we performed promoter deletion analyses. Strains bearing promoter deletion constructs were induced to form filaments in YEPD plus 10% serum at 37°C, Spider medium (nitrogen and carbon starvation) and/or Lee’s medium pH 6.8 (neutral pH) and reporter gene expression was measured. In the *NRG1* promoter we identified several distinct condition-specific response elements for YEPD plus 10% serum at 37°C and Spider medium. In the *UME6* promoter we also identified response elements for YEPD plus 10% serum at 37°C. While a few of these elements are distinct, others overlap with those which respond to Lee’s pH 6.8 medium. Consistent with *UME6* possessing a very long 5’ UTR, many response elements in the *UME6* promoter are located significantly upstream from the coding sequence. Our data indicate that certain distinct condition-specific elements can control expression of *C*. *albicans UME6* and *NRG1* in response to key filament-inducing environmental cues. Because *C*. *albicans* encounters a variety of host microenvironments during infection, our results suggest that *UME6* and *NRG1* expression can be differentially modulated by multiple signaling pathways to control filamentation and virulence *in vivo*.

## Introduction

*Candida albicans* is an opportunistic human fungal pathogen and a significant cause of disease in immunocompromised individuals such as AIDS patients, organ transplant recipients and cancer patients on chemotherapy [1–4]. It is estimated that 70% of women will experience at least one episode of vulvovaginal candidiasis in their lifetime [5, 6]. In addition to cutaneous and mucosal infections, *C*. *albicans* can disseminate and cause life-threatening, systemic infections [1, 7]. With a mortality rate of ~40%, *Candida* species are the fourth leading cause of hospital-acquired bloodstream infections in the U.S. [8, 9].

While several traits contribute to the pathogenesis of *C*. *albicans*, morphogenesis, the ability to transition between oval-shaped budding yeast cells and filaments (elongated cells attached end-to-end), is an essential virulence property of this organism [1, 7, 10]. Filamentation plays an important role in tissue invasion, cell damage, biofilm formation, thigmotropism as well as the ability to escape from and lyse macrophages [11–17]. Furthermore, genes involved in the physical process of filamentous growth are co-regulated with genes important for other virulence processes, including adhesion and degradation of host cell membranes [18, 19]. Several previous studies have clearly demonstrated that the ability of *C*. *albicans* to undergo a reversible transition from yeast to filaments is required for virulence in a mouse model of systemic candidiasis [13, 15, 20–24].

A variety of host environmental cues are known to trigger the *C*. *albicans* yeast-to-filament transition, including 37°C, serum, human hormones, starvation and neutral pH [25–27]. These host conditions activate signal transduction pathways (eg: MAP kinase and cAMP-PKA pathways) resulting in the induction of filament-specific genes [28]. Several *C*. *albicans* transcriptional regulators are known to play critical roles in this process. Importantly, two of these regulators, Nrg1 and Ume6, are themselves controlled at the transcriptional level by filament-inducing conditions. Nrg1, a zinc finger DNA-binding protein, functions (via recruitment of the Tup1 corepressor) as a key transcriptional repressor of filament-specific genes under non-filament-inducing conditions [21, 22]. In the presence of certain filament-inducing conditions (eg: growth in serum at 37°C) the *NRG1* transcript is down-regulated, causing the expression of filament-specific genes. In addition, the Nrg1 repressor is transiently displaced from hyphal-specific promoters via activation of the cAMP-PKA pathway in the presence of serum at 37°C [29]. The *nrg1*Δ/Δ mutant is avirulent and constitutive high-level expression of *NRG1* has also been shown to block the yeast-to-filament transition, leading to highly attenuated virulence in a mouse model of systemic candidiasis [15, 21, 22].

Ume6, also a zinc finger DNA-binding protein, is a filament-specific transcriptional regulator that is required for hyphal extension [30]. A variety of different environmental filament-inducing conditions, including growth at 37°C in the presence of serum, Spider medium and Lee’s medium, pH 6.8 (neutral pH), are known to induce the *UME6* transcript. *UME6* is also a downstream target of multiple filamentous growth transcriptional regulators [31]. Both the level and duration of *UME6* expression are important for determining *C*. *albicans* morphology and Ume6 protein stability is controlled by both oxygen- and CO_2_-sensing pathways [24, 32]. The *ume6*Δ/Δ mutant is attenuated for virulence and constitutive high-level expression of *UME6* increases hyphal formation *in vivo* and is sufficient to promote virulence in a mouse model of systemic candidiasis [24, 30]. We have previously shown that both *NRG1* and *UME6* function together in a feedback loop to promote filament-specific gene expression in the presence of strong filament-inducing conditions [30].

While the critical roles that both *UME6* and *NRG1* play in regulating both the *C*. *albicans* yeast-filament transition and virulence have been well-characterized, considerably less is known about the exact mechanism(s) by which host environmental signals control expression of these key regulators at the transcriptional level. In this study we characterize the promoters of both *NRG1* and *UME6* and identify elements that respond to specific filament-inducing conditions. We also determine the extent to which these elements and, by inference, the upstream signaling pathways which target these elements, overlap. Overall, our results provide new and important information about how environmental cues, similar to those encountered in the host, regulate the expression of two key transcription factors that govern filamentation and virulence in the major human fungal pathogen *C*. *albicans*.

## Materials and Methods

### Media and growth conditions

YEPD (yeast-extract peptone dextrose) medium at 30°C was used as a standard non-filament-inducing condition [33]. Preparation of Spider and Lee’s media were performed as described previously [34, 35]. Induction of filamentation by growth in YEPD + 10% serum at 37°C, Spider medium at 30°C and Lee’s medium pH 6.8 at 30°C was conducted as described previously [30] with modifications. Strains were grown overnight in YEPD at 30°C to OD_600_ ~ 4.0 and then diluted 1:10 into pre-warmed YEPD + 10% serum at 37°C and 1:20 into Spider medium at 30°C. For Lee’s medium inductions, 10 mL of an overnight culture of cells grown in YEPD at 30°C was centrifuged, washed and resuspended in 5 mL 1X PBS. 2.5 mL of this cell suspension was inoculated into pre-warmed Lee’s pH 6.8 or Lee’s pH 4.5 medium at 30°C. Cells were harvested 30 minutes following induction in serum medium at 37°C, 6 hours following induction in Spider medium at 30°C and 1 hour following induction in Lee’s pH 6.8 medium for total RNA isolation. To determine the effect of farnesol on *lacZ* reporter gene expression, strains were grown overnight in YEPD medium at 30°C to OD_600_ ~ 4.0. Cells were then diluted 1:10 into pre-warmed YEPD at 30°C in the presence and absence of 40 μM farnesol (Sigma-Aldrich; St. Louis, MO). Aliquots of cells were harvested from both the initial overnight culture as well as the diluted cultures (30 minutes following dilution) for RNA extraction.

### Strain and DNA constructions

*NRG1* promoter deletion strains were constructed as follows: varying regions of the *NRG1* upstream intergenic region were amplified by PCR from *C*. *albicans* SC5314 genomic DNA using primers 1–16 (please see S1 Table and S2 Table for complete listings of strains and primers, respectively, used in this study). PCR products were digested with PstI-XhoI and cloned into plasmid placbasal [36]. The resulting promoter fragment-placbasal constructs were linearized at *RPS1* by digestion with StuI and transformed into strain CAI4 to generate the *NRG1* promoter deletion strains. The pUME6_4.9–7 kb UR—_lac, pUME6_4.9–6 kb UR—_lac, pUME6_4–6 kb UR—_lac and pUME6_3–6 kb UR—_lac plasmids were constructed as follows: each respective region of the *UME6* upstream intergenic region was amplified by PCR using primers 20, 21, and 27–29. PCR products were digested with PstI-SphI and cloned into plasmid placbasal. To generate the 5.5 kb and 5 kb *UME6* promoter deletion strains, regions from -1 bp to -2541 bp, -632 bp to -5494 bp and -632 bp to -4976 bp were amplified by PCR with primer sets 17/19, 24/25 and 25/26, respectively. All three fragments were digested with PstI-HindIII and cloned separately into pBS [37]. The -1 bp to -2077 bp fragment was released from pBS by digesting with HindIII-SphI and the -2077 bp to -5494 bp and -2077 bp to -4976 bp fragments were released from pBS by digesting with PstI-HindIII. A 3-piece ligation was used to clone the -1 bp to -2077 bp fragment and -2077 bp to -5495 bp fragment into plasmid placbasal to generate the pUME6_5.5 kb UR-_lac plasmid. This method was also used to clone the -1 bp to -2077 bp fragment and -2077 bp to -4976 bp fragment into plasmid placbasal to generate the pUME6_5 kb UR—_lac plasmid. The pUME6_7 kb UR—_lac plasmid was constructed by amplifying the following fragments by PCR: -1801 bp to -5824 bp with primers 18/23 and -5491 bp to -7009 bp with primers 21/22. The -1 bp to -2077 bp fragment was prepared as described above. The fragment from -1801 bp to -5824 bp was digested with XhoI-HindIII and cloned into pBS and the fragment from -5491 bp to -7009 bp was digested with PstI-XhoI and cloned into pBS separately. The fragments from -2077 bp to -5808 bp and from -5808 bp to -7009 bp were released from pBS by XhoI-HindIII and PstI-XhoI digests, respectively. A 4-piece ligation was used to clone the three fragments generated above into placbasal to create plasmid pUME6_7 kb UR—_lac. Plasmid pUME6_6kb UR—_lac has been described previously [38]. All *UME6* promoter deletion plasmids and the plac plasmid were linearized at *RPS1* by digestion with StuI and transformed into strain CAI4 to generate the *UME6* promoter deletion strains. All promoter fragments were sequenced prior to transformation into strain CAI4. Integration events were confirmed by whole-cell PCR using primers which flank the integration sites.

### RNA preparation

RNA for qRT-PCR analysis was prepared as described previously [38].

### Quantitative RT-PCR analysis

cDNA for qRT-PCR analysis was prepared from total RNA as described previously [38], except that DNase from Life Technologies, Inc. (Carlsbad, CA) was used. qRT-PCR analysis was performed as described previously [38] using primer pairs 30/31 for *lacZ* and 32/33 for *ACT1* with an annealing temperature of 57.4°C. The Pfaffl method was used to normalize expression levels of *lacZ* to those of an internal *ACT1* control[39]. All qRT-PCR reactions were performed in biological duplicate and technical duplicate. Values shown indicate mean ± SEM.

## Results

### Defining the *NRG1* and *UME6* promoter regions

**Fig 1:**
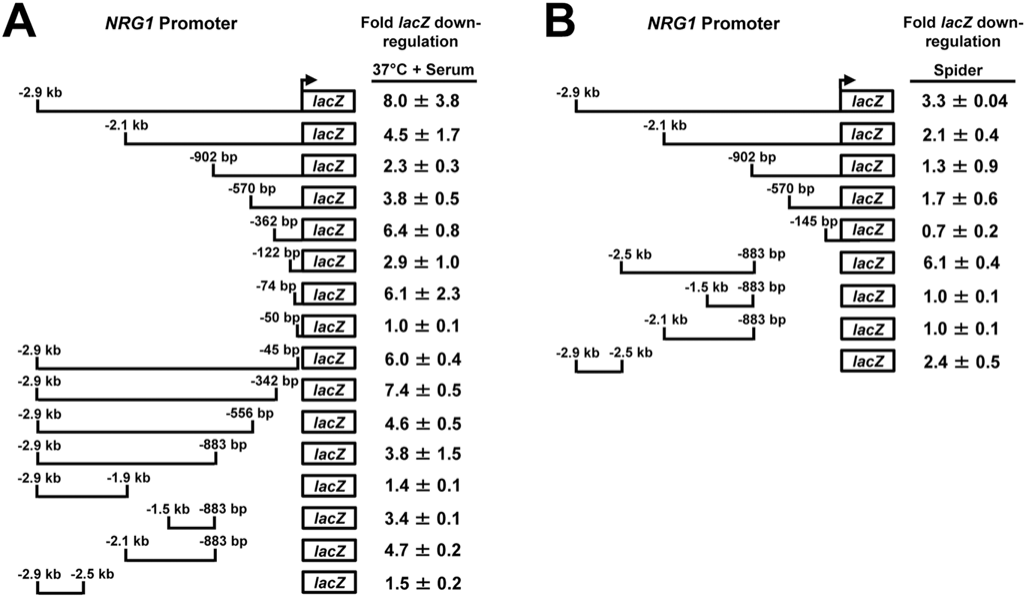
Deletion analysis of the *NRG1* promoter identifies specific response elements for serum at 37°C and Spider. Strains bearing the indicated *NRG1* promoter-*lacZ* reporter constructs were grown under both non-filament-inducing (YEPD at 30°C) and filament-inducing (YEPD + 10% serum at 37°C or Spider at 30°C) conditions. Cells were harvested from both non-inducing and filament-inducing cultures for total RNA isolation and cDNA synthesis. For the serum and temperature induction experiment (A) cells were harvested at the 30 minute post-induction time point and for the Spider induction experiment (B) cells were harvested at the 6 hr. post-induction time point. *lacZ* expression was determined by qRT-PCR and normalized to *ACT1* levels. Fold down-regulation of *lacZ* was determined by dividing normalized *lacZ* values obtained from cells grown under non-filament inducing conditions by normalized *lacZ* values obtained from cells induced to form filaments under each condition.

In order to identify the *NRG1* promoter, we cloned 902 bp, 2.1 kb and 2.9 kb fragments of the *NRG1* upstream intergenic region in a *Streptococcus thermophilus lacZ* reporter construct [36] which was subsequently integrated at the *C*. *albicans RPS1* locus. Each strain was grown under both non-filament-inducing (YEPD at 30°C) as well as strong filament-inducing (YEPD plus 10% serum at 37°C) conditions. We observed the strongest level of *lacZ* down-regulation (8-fold) in response to filament induction with the strain containing the 2.9 kb *NRG1* upstream intergenic region reporter construct (Fig. 1A). This level of *NRG1* down-regulation was similar, if not slightly greater than, that observed with the natural transcript upon serum and temperature induction [18, 40]. In addition, an *in silico* analysis of the *NRG1* 3 kb upstream region using Transcription Element Search Software (www.cbil.upenn.edu/cgi-bin/tess/tess) (default parameters) [41] identified two putative binding sites each for Rim101, a pH-responsive transcriptional regulator of filamentous growth, and Cph1, a regulator of *C*. *albicans* mating and morphogenesis. Previous studies have also identified binding sites in this region for other transcriptional regulators of morphogenesis including Ndt80 and Efg1 [42, 43]. Taken together, these results suggest that the *NRG1* promoter is located within the 2.9 kb upstream region.

We have recently demonstrated that a 6 kb *UME6* upstream region is required for *C*. *albicans* filamentation in response to serum at 37°C [38]. In addition, a fragment containing this region was sufficient to direct strong activation of the heterologous *S*. *theromphilus lacZ* reporter in response to growth in serum at 37°C. These results suggested that critical *UME6* promoter elements are located in this region. However, to ensure that we did not overlook important, but non-essential *UME6* promoter elements, we conducted an *in silico* analysis (as described above) for 7 kb of the *UME6* upstream intergenic region and identified putative binding sites for a variety of key *C*. *albicans* filamentous growth transcriptional regulators including Rim101, Cph1 and Fkh2. Results of this analysis are consistent with those of a previous study showing that *cph1*Δ/Δ and *fkh2*Δ/Δ deletion strains are defective for *UME6* induction in response to hyphal-inducing conditions [31]. Previous studies using ChIP-Chip and DNA tiling array analysis have also described binding sites for Ndt80, Efg1, Nrg1, Brg1 and Hms1 in the *UME6* upstream region [42–45]. Importantly, several of the binding sites were located in the 6 to 7 kb *UME6* upstream region. In addition, a number of the factors which bind to these sites have been shown to control *UME6* expression [30, 31, 44, 45]. We have also recently demonstrated that the *UME6* transcript has an exceptionally long 3 kb 5’ UTR which functions in translational inhibition [38]. These previous results, combined with our *in silico* analysis, suggest that the *UME6* promoter is located in the 7 kb upstream region.

### Identification of filament condition-specific response elements in the *NRG1* promoter

In order to determine which elements in the *NRG1* promoter are important for controlling expression in response to specific filament-inducing conditions, we conducted a promoter deletion analysis. Various deletions of the *NRG1* promoter were fused to a *S*. *thermophilus lacZ* reporter [36] and integrated at the *C*. *albicans RPS1* locus. *lacZ* expression was measured using qRT-PCR under both non-inducing conditions (YEPD at 30°C) as well as several known filament-inducing conditions. Time points used to harvest cells for each filament-inducing condition used in the deletion analyses described in this study were specifically chosen to observe maximal transcriptional effects (ie: *NRG1* down-regulation and *UME6* induction) based on pilot experiments.

To examine the response to serum and temperature, strains carrying *NRG1* promoter deletions were induced to form filaments in YEPD + 10% serum at 37°C. As noted previously, we observed ~8-fold down-regulation of *lacZ* expression in the reporter strain carrying the full-length -2.9 kb *NRG1* promoter fragment in response to growth in serum at 37°C (Fig. 1A). In general, 5’ deletions of the *NRG1* promoter to the -122 bp position caused mild reductions in the level of down-regulation. Deletion of the -362 bp to -122 bp region led to a significant decline in *lacZ* down-regulation suggesting the presence of a serum and temperature responsive negative element. However, a deletion from -122 bp to -74 bp led to enhanced *lacZ* repression in response to 37°C + serum, indicating that the deleted sequence may contain a positive element. Importantly, -74 bp of the *NRG1* promoter was the minimum region necessary to down-regulate the *lacZ* reporter in response to 37°C + serum. The -50 bp promoter did not drive basal *lacZ* expression under non-inducing conditions, which suggested that the region from -50 bp to -74 bp contains a negative response element. We also determined, using 5’ and 3’ *NRG1* promoter deletion constructs, that the region between -883 bp and -1.5 kb contains another key temperature- and serum-responsive negative element (Fig. 1A).

In order to identify elements important for down-regulation of *NRG1* in response to nitrogen and carbon starvation, strains bearing *NRG1* promoter deletion constructs were induced to form filaments in Spider medium at 30°C. These strains were also grown under non-inducing conditions, YEPD at 30°C, as a control. We observed that the full-length 2.9 kb *NRG1* promoter down-regulated *lacZ* expression ~3.3-fold in response to growth in Spider medium at 6 hrs. (Fig. 1B). Spider is a weaker filament-inducing condition and the 6 hr. time point was chosen for further analysis because the greatest *NRG1* down-regulation was observed at this post-induction time point. While a 5’ deletion containing 2.1 kb of the *NRG1* promoter directed ~2.1-fold *lacZ* down-regulation, additional 5’ promoter deletions were unable to significantly reduce *lacZ* expression. We next used 3’ *NRG1* promoter deletion strains to further localize elements important for *NRG1* down-regulation in response to Spider medium (Fig. 1B). The region from -883 bp to -2.5 kb down-regulated *lacZ* expression ~6.1-fold. However, other promoter deletion constructs in that region (-883 bp to -2.1 kb and -883 bp to -1.5 kb) did not significantly down-regulate *lacZ* expression. Interestingly, we also observed that the -2.5 kb to -2.9 kb region was sufficient to direct > 2-fold *lacZ* down-regulation. Taken together, these results suggest that the *NRG1* promoter region from -2.1 kb to -2.9 kb contains two Spider condition-specific negative elements; the first element is located between -2.1 kb and -2.5 kb and the second element is between -2.5 kb and -2.9 kb.

### Response of the *NRG1* promoter to hyphal shock

**Fig 2:**
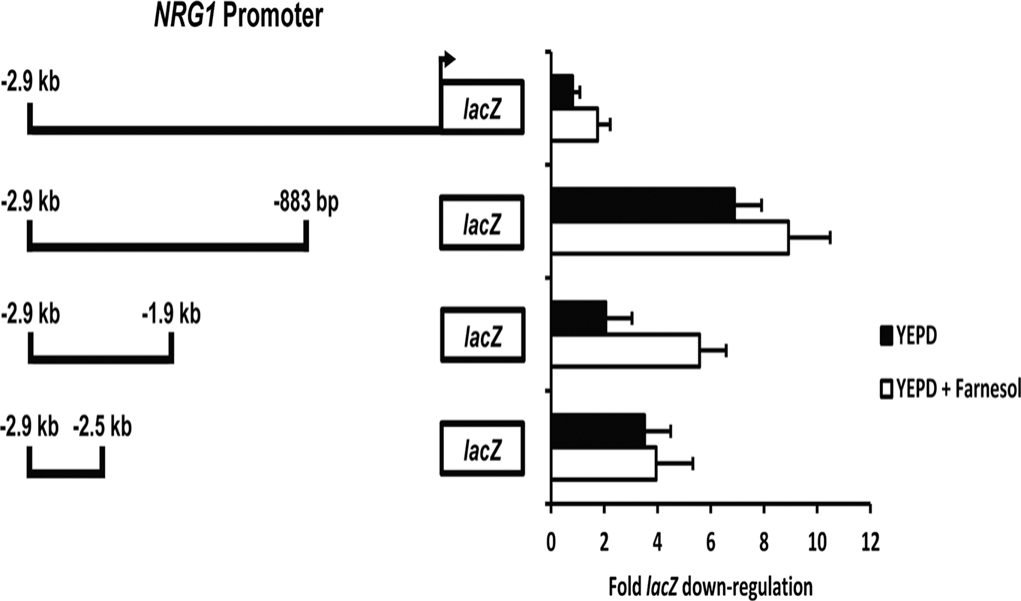
Identification of hyphal shock response elements in the *NRG1* promoter. Strains bearing the indicated *NRG1* promoter-*lacZ* reporter constructs were grown overnight under non-inducing conditions (YEPD at 30°C) and diluted into fresh YEPD medium in the presence or absence of 40 μM farnesol at 30°C. Cells were harvested from both the overnight culture (zero time point) as well as at 30 minutes following dilution for total RNA isolation and cDNA synthesis. *lacZ* expression values were determined by qRT-PCR and normalized to *ACT1* levels. Fold down-regulation of *lacZ* was determined by dividing normalized *lacZ* values from cells grown overnight under non-filament inducing conditions (zero time point) by normalized *lacZ* values from cells diluted into fresh YEPD or YEPD plus 40 μM farnesol.

We have previously described a “hyphal shock” phenomenon whereby *C*. *albicans* cells can be induced to form filaments when diluted from a saturated overnight culture into non-inducing media, such as YEPD at 30°C [18]. In our reporter assays we routinely harvest cells for RNA preparation prior to induction at the zero hour time point to examine for this effect. Interestingly, while the full-length *NRG1* promoter did not show down-regulation in response to dilution in YEPD at 30°C, we did observe that certain *NRG1* promoter deletion constructs showed down-regulation of *lacZ* expression at 30 minutes growth in YEPD at 30°C following dilution from the zero time point overnight culture (Fig. 2). Because hyphal shock is believed to be mediated by farnesol, a quorum-sensing molecule that has specifically been shown to control Nrg1 protein stability [46], we decided to further investigate this phenomenon and define “hyphal shock”-sensitive regions within the *NRG1* promoter. Unlike the full-length 2.9 kb *NRG1* promoter reporter, we observed that the -883 bp to -2.9 kb *NRG1* promoter deletion reporter was particularly sensitive to dilution into fresh YEPD, resulting in ~7-fold down-regulation of *lacZ*. This finding suggested that the -1 bp to -883 bp region is important for activation of *NRG1* in response to hyphal shock. In contrast, both the -1.9 kb to -2.9 kb and -2.5 kb to -2.9 kb fragments directed ~2–4-fold *lacZ* down-regulation when cells were diluted in fresh medium, suggesting that the -883 bp to -1.9 bp and -2.5 kb to -2.9 kb regions are important for repression of *NRG1* in response to hyphal shock.

As discussed above, one hypothesis is that *C*. *albicans* filamentation which occurs during hyphal shock is the result of a rapid release from filament inhibition by quorum-sensing molecules, such as farnesol [47]. Farnesol inhibits cAMP signaling, thus favoring yeast growth [29, 48]. Dilution of cells at higher optical densities into fresh media would also dilute overall levels of farnesol and other small molecules. We speculated that the sudden loss of farnesol-mediated inhibition of the cAMP pathway might affect transcript levels directed by the *NRG1* promoter. In order to test this hypothesis, strains carrying *NRG1* promoter deletions were grown overnight in YEPD at 30°C and diluted into fresh YEPD at 30°C in the presence and absence of 40 μM farnesol. Cells were harvested following 30 minutes growth for total RNA isolation. Interestingly, we observed that growth in farnesol did not prevent down-regulation of *lacZ* expression directed by the *NRG1* promoter deletion constructs (Fig. 2). These results suggest that farnesol-mediated inhibition of the cAMP pathway does not play an important role in regulating the *NRG1* promoter response to hyphal shock.

### Identification of filament condition-specific regulatory regions in the *UME6* promoter

Similar to *NRG1*, *UME6* also encodes a transcription factor which itself is controlled at the transcriptional level by filament-inducing conditions [30, 31]. In order to identify DNA elements in the *UME6* promoter important for filament condition-specific control, various *UME6* promoter deletions were fused to a *lacZ* reporter and integrated at the *C*. *albicans RPS1* locus. We examined *lacZ* expression of these strains using qRT-PCR when cells were grown under several known filament-inducing conditions.

**Fig 3:**
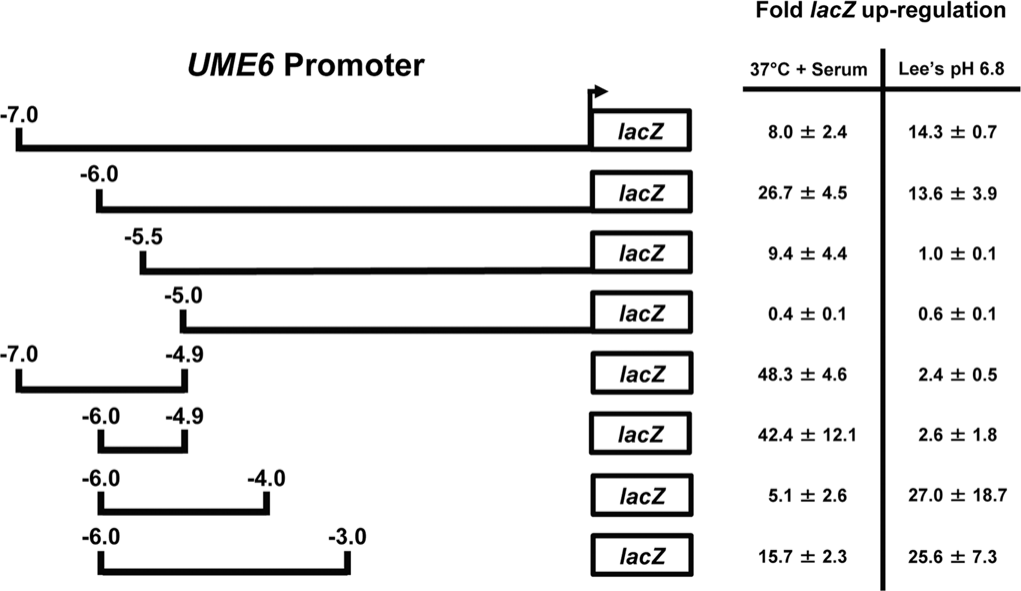
Deletion analysis of the *UME6* promoter identifies condition-specific response elements. Strains bearing the indicated *UME6* promoter-*lacZ* reporter constructs were grown under non-inducing conditions (YEPD at 30°C for the serum and temperature induction experiment and Lee’s pH 4.5 medium for the neutral pH induction experiment) and the indicated filament-inducing conditions. Cells were harvested (at 30 minutes for the serum and temperature induction experiment and at 1 hour for the neutral pH induction experiment) for total RNA isolation and cDNA synthesis. *lacZ* expression values were determined by qRT-PCR and normalized to *ACT1* levels. Fold up-regulation of *lacZ* was determined by dividing normalized *lacZ* values from cells induced to form filaments under each filament-inducing condition by normalized *lacZ* values from cells grown under non-filament inducing conditions. Please note that the fold *lacZ* up-regulation value for the—6.0 kb *UME6* promoter construct in 37°C + serum has been reported previously [38].

We first sought to identify *UME6* promoter elements important for transcriptional induction in response to growth in 10% serum at 37°C. Cells were harvested 30 minutes following filament induction under these conditions and total RNA was prepared for qRT-PCR analysis. We previously observed that a 6 kb *UME6* promoter fragment directed strong (~27-fold) induction of *lacZ* expression in response to serum and temperature [38]. Interestingly, increasing the *UME6* promoter length to 7 kb reduced *lacZ* up-regulation to ~8-fold, suggesting that this region contains a serum and temperature-responsive negative element (Fig. 3). A 5’ *UME6* promoter deletion analysis indicated that *lacZ* induction was abolished in the strain expressing the 5.0 kb, but not the 5.5 kb *UME6* promoter construct, suggesting that this region is required for the serum and temperature response. In addition, induction was decreased in the strain expressing the 5.5 kb vs. 6.0 kb *UME6* promoter indicating the presence of a positive response element in this region. We also examined the response of 3’ *UME6* promoter deletions during a serum and temperature induction. A deletion strain bearing -4.0 kb to -6.0 kb of the *UME6* promoter generated a low level induction of *lacZ* (~5-fold) (Fig. 3). An additional deletion of the -4 kb to -4.9 kb region resulted in ~40-fold up-regulation of *lacZ* expression. This result suggests that the -4 kb to -4.9 kb *UME6* promoter region contains a negative element for serum and temperature. Finally, the -3.0 kb to -6.0 kb region showed greater induction compared to the -4.0 kb to -6.0 kb region suggesting the presence of a serum- and temperature-responsive positive element between -3.0 kb and -4.0 kb.

In order to identify neutral pH responsive *UME6* promoter elements, strains carrying *UME6* promoter deletions were induced to form filaments in Lee’s pH 6.8 at 30°C (Lee’s pH 4.5 at 30°C was used as a non-inducing control). Cells were harvested 1 hour following induction for total RNA isolation and qRT-PCR analysis. We observed ~14-fold *lacZ* induction with both the 7 kb and 6 kb *UME6* promoters (Fig. 3). Interestingly, *lacZ* was not induced by the 5.5 kb promoter, suggesting that the -5.5 kb to -6 kb region is necessary for *lacZ* up-regulation in response to neutral pH. We also observed mild *lacZ* induction with the -4.9 kb to -7 kb and -4.9 kb to -6 kb *UME6* promoter deletions. In contrast to our previous observations regarding the *UME6* promoter element response to serum and temperature, the -4.0 kb to -6.0 kb and -3.0 kb to -6.0 kb deletions led to significantly increased *lacZ* induction in response to neutral pH compared to the other 3’ *UME6* promoter deletions. These results suggest the presence of a positive neutral pH response element in the -4.0 kb to -4.9 kb promoter region (Fig. 3). Overall, our results suggest that specific regions of the *UME6* promoter control responses to serum at 37°C and neutral pH.

## Discussion

**Fig 4:**
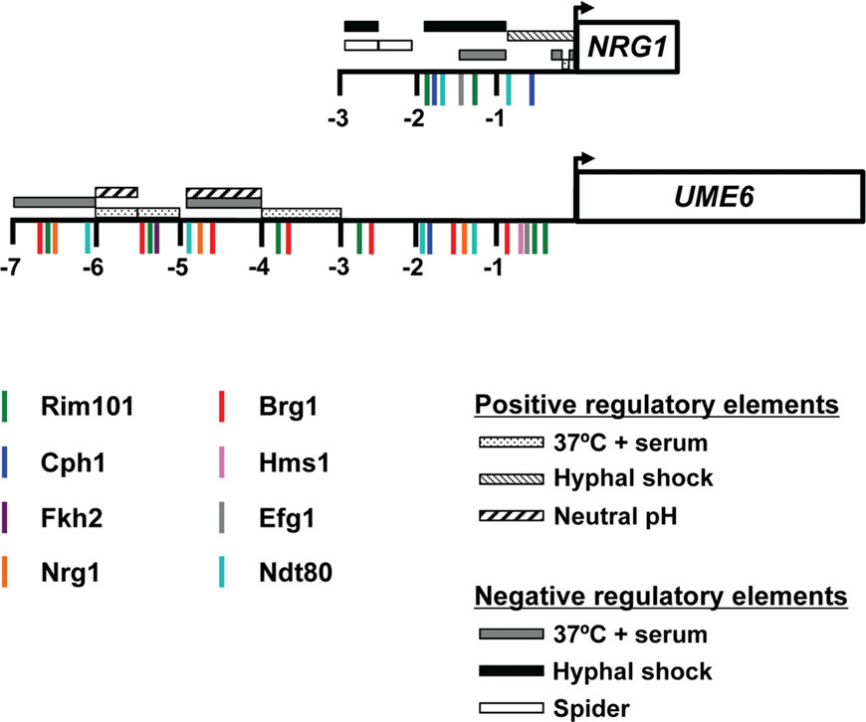
Summary of filament condition-specific response regions in the *NRG1* and *UME6* promoters. Schematic representation of the *NRG1* and *UME6* promoters showing specific regions important for responding to the indicated filament-inducing conditions. For each promoter, putative transcription factor binding sites identified by Transcription Element Search Software (TESS, (www.cbil.upenn.edu/cgi-bin/tess/tess, default parameters [41]) (Rim101, Cph1 and Fkh2) and by previous studies (Nrg1) [44] are shown. Transcription factor binding sites identified in previous studies by ChIP-Chip and DNA tiling array analysis (Brg1, Hms1, Efg1 and Ndt80) are also included [42–45]. Please note that *S*. *cerevisiae* consensus binding sequences for orthologs of Fkh2 and Cph1 were used in our analysis; Forkhead protein binding sites are well-conserved among eukaryotes[52]. Numbers indicate position (in kb) upstream from the start codon. Note: not drawn exactly to scale.

Nrg1 and Ume6 represent key transcriptional regulators of the *C*. *albicans* yeast-filament transition which function together in a feedback loop under filament-inducing conditions and are themselves transcriptionally controlled [21, 22, 30, 31]. Characterization of the *NRG1* and *UME6* promoter regions and identification of elements in these regions which respond to specific filament-inducing conditions (Fig. 4) therefore represents an important step in determining how various *C*. *albicans* filamentous growth signaling pathways ultimately control the yeast-filament transition and virulence at the transcriptional level. In the case of the *NRG1* promoter, we have identified a key serum and temperature-responsive repressor element in the -883 bp and -1.5 kb region. This region also contains potential binding sites for Ndt80 and Efg1 (Fig. 4). Efg1, important for induction of filament-specific gene expression in response to serum and temperature, is a downstream target of the cAMP/PKA pathway [49–51]. Efg1 has also previously been shown to be specifically important for down-regulation of the *NRG1* transcript under these conditions [21]. However, we cannot exclude the possibility that additional transcriptional regulators, which have not yet been identified, also function to down-regulate *NRG1* via the -1.5 kb to -883 bp or -74 bp to -50 bp regions. This is especially the case for the later region since potential binding sites for known *C*. *albicans* transcription factors were not identified in this region by our *in silico* analysis (Fig. 4). Similarly, the *in silico* analysis did not identify potential binding sites for known transcription factors in the Spider responsive elements, suggesting that down-regulation of *NRG1* under this condition most likely occurs via regulators which are currently uncharacterized.

While numerous attempts were made to identify pH-responsive elements that control *NRG1* expression, we were unable to detect down-regulation of the full-length *NRG1* promoter-*S*. *thermophilus lacZ* reporter in α-MEM pH 7.4, as previously reported [53], or in Lee’s medium, pH 6.8 (data not shown). Although our *in silico* analysis identified two potential Rim101 binding sites within the *NRG1* promoter (Fig. 4), previous work has suggested that *NRG1* and *RIM101* function in independent pathways [54]. One possible explanation is that additional elements located greater than 2.9 kb upstream of the start codon are required for *NRG1* down-regulation in response to neutral pH. Alternatively, secondary structure of the native chromatin at the *NRG1* locus, which our reporter does not replicate, may also play an important role in pH regulation of *NRG1* transcription.

This study is the first to identify DNA elements important for the ability of *C*. *albicans* to regulate gene expression in response to hyphal shock which has previously been shown to induce filamentation [18, 47]. Our finding that farnesol fails to down-regulate the *NRG1* promoter is consistent with previous studies indicating that *NRG1* transcript expression and down-regulation are not affected by this quorum-sensing molecule [46, 55]. Interestingly, however, a recent study has shown that farnesol blocks degradation of the Nrg1 protein, leading to yeast growth [46]. At this point we cannot exclude the possibility that other quorum-sensing molecules may play an important role in the hyphal shock transcriptional response at the *NRG1* promoter. In addition, since hyphal shock was induced by diluting late log phase cultures into fresh nutrient-rich medium, signaling pathways involved in nutrient sensing, such as Tor, might also be involved in this process.

Several of our findings regarding regulatory regions in the *UME6* promoter are also consistent with the location of known filamentous growth transcriptional regulator binding sites. More specifically, we have identified the -4.0 kb to -4.9 kb region as containing a key serum and temperature responsive negative element and this region has multiple predicted Nrg1 binding sites at ~ -4800 bp that might contribute towards the observed transcriptional repression [44]. This observation is consistent with our previous finding that *UME6* is under negative control by Nrg1 [30]. In addition, the -5 kb to -5.5 kb *UME6* promoter region required for induction contains a Brg1 binding site at ~ -5.3 kb [44]. Brg1 has previously been shown to be required for *UME6* expression during growth in YEPD at 37°C with rapamycin [44]. While there are several predicted Rim101 binding sites throughout the *UME6* promoter, none of these sites are located in the -5.5 kb to -6 kb or -4.0 to -4.9 kb neutral pH response regions. It is possible that other pH-responsive transcription factors, which have not yet been identified, may be controlling *UME6* expression through these elements.

One limitation of our study is that growth in YEPD at 30°C, a non-physiological temperature, was used as a non-filament-inducing condition. We chose to use YEPD at 30°C as the non-inducing condition because growth in YEPD at 37°C causes mild filamentation as well as transcriptional effects, such as induction of *UME6*, that could significantly blunt the sensitivity of our assay [18, 30]. However, a limitation of this approach is that we cannot exclude the possibility that our observed results are due to a temperature shift rather than specific changes in *UME6* or *NRG1* expression.

It is important to bear in mind that *C*. *albicans* filamentation and the expression of Nrg1 and Ume6 are controlled at multiple levels. As previously mentioned, Nrg1 is regulated at the level of protein stability in response to farnesol [46]. An anti-sense mRNA stability mechanism is also known to control *NRG1* [56]. In addition, we have recently shown that *UME6* expression is inhibited by a 5’ UTR-mediated translational efficiency mechanism [38] and another recent report indicates that Ume6 protein levels are synergistically stabilized by hypoxia and high CO_2_ [32]. In this study, we provide new information specifically regarding the transcriptional control of these important *C*. *albicans* filamentous growth regulators. Our identification of distinct filament condition-specific response elements in both the *NRG1* and *UME6* promoters suggests that a complex array of multiple filamentous growth signaling pathways either directly or indirectly target these key regulators. While several signaling pathways have been previously characterized, additional pathways remain unknown and it is hoped that future studies will shed more light in this area.

## Conclusions

Nrg1 and Ume6 are key transcriptional regulators of filament-specific gene expression and virulence in *C*. *albicans*. However, mechanisms controlling the expression of these regulators in response to filament-inducing conditions are poorly understood. In this study, we have identified several condition-specific response elements within the *NRG1* and *UME6* promoters for some of the most commonly investigated filament-inducing conditions, including growth in serum at 37°C, Spider medium (nitrogen and carbon starvation) and neutral pH (summarized in Fig. 4). Several of these elements contain predicted binding sites for known transcriptional regulators of filamentation which have been shown to be important for controlling *NRG1* and/or *UME6* expression. Other response elements appear to be targeted by signaling pathways and/or transcriptional regulators which have not yet been fully characterized. Overall, because *C*. *albicans* encounters a variety of microenvironments during infection that can promote or inhibit filamentation, our results suggest that *UME6* and *NRG1* transcripts can be differentially modulated by multiple signaling pathways which respond to host environmental cues. This modulation, in turn, could potentially affect virulence and morphology determination in a niche-specific manner *in vivo*.

## Supporting Information

S1 Table Strains used in this study. (PDF)

S2 Table Primers used in this study. (PDF)

## References

[1] Odds FC. *Candida* and Candidosis. 2nd ed London: Baillière Tindall; 1988 468 p.

[2] Filler SG, Kullberg BJ. Deep-seated candidal infections In: Calderone R, editor. *Candida* and candidiasis. Washington, D.C.: ASM Press; 2002 p. 341–8.

[3] Cannon RD, Chaffin WL. Oral colonization by *Candida albicans* . Crit Rev Oral Biol Med. 1999;10(3):359–83. Epub 2000/04/12. 10759414 10.1177/10454411990100030701

[4] Dongari-Bagtzoglou A, Wen K, Lamster IB. *Candida albicans* triggers interleukin-6 and interleukin-8 responses by oral fibroblasts in vitro. Oral Microbiol Immunol. 1999;14(6):364–70. Epub 2000/07/15. 10895692 10.1034/j.1399-302x.1999.140606.x

[5] Fidel PL Jr, Vazquez JA, Sobel JD. *Candida glabrata*: review of epidemiology, pathogenesis, and clinical disease with comparison to *C*. *albicans* . Clin Microbiol Rev. 1999;12(1):80–96. 9880475 10.1128/cmr.12.1.80 PMC88907

[6] Sobel JD. Pathogenesis and treatment of recurrent vulvovaginal candidiasis. Clin Infect Dis. 1992;14 Suppl 1:S148–53. Epub 1992/03/01. 1562688 10.1093/clinids/14.supplement_1.s148

[7] Calderone RA, Clancy CJ, editors. *Candida* and Candidiasis. 2nd ed Washington, D.C.: ASM Press; 2012.

[8] Edmond MB, Wallace SE, McClish DK, Pfaller MA, Jones RN, Wenzel RP. Nosocomial bloodstream infections in United States hospitals: a three-year analysis. Clin Infect Dis. 1999;29(2):239–44. 10476719 10.1086/520192

[9] Wisplinghoff H, Bischoff T, Tallent SM, Seifert H, Wenzel RP, Edmond MB. Nosocomial bloodstream infections in US hospitals: analysis of 24,179 cases from a prospective nationwide surveillance study. Clin Infect Dis. 2004;39(3):309–17. Epub 2004/08/13. doi: 10.1086/421946 CID32752 [pii] 15306996 10.1086/421946

[10] Brown AJ. Expression of growth form-specific factors during morphogenesis in *Candida albicans* In: Calderone RA, editor. *Candida* and Candidiasis. Washington, D.C.: ASM Press; 2002 p. 87–93.

[11] Kumamoto CA, Vinces MD. Contributions of hyphae and hypha-co-regulated genes to *Candida albicans* virulence. Cellular microbiology. 2005;7(11):1546–54. 16207242 10.1111/j.1462-5822.2005.00616.x

[12] Korting HC, Hube B, Oberbauer S, Januschke E, Hamm G, Albrecht A, et al Reduced expression of the hyphal-independent *Candida albicans* proteinase genes *SAP1* and *SAP3* in the *efg1* mutant is associated with attenuated virulence during infection of oral epithelium. J Med Microbiol. 2003;52(Pt 8):623–32. 12867554 10.1099/jmm.0.05125-0

[13] Lo HJ, Kohler JR, DiDomenico B, Loebenberg D, Cacciapuoti A, Fink GR. Nonfilamentous *C*. *albicans* mutants are avirulent. Cell. 1997;90(5):939–49. 9298905 10.1016/s0092-8674(00)80358-x

[14] Gow NA, Brown AJ, Odds FC. Fungal morphogenesis and host invasion. Current opinion in microbiology. 2002;5(4):366–71. Epub 2002/08/06. doi: S1369527402003387 [pii] 12160854 10.1016/s1369-5274(02)00338-7

[15] Saville SP, Lazzell AL, Monteagudo C, Lopez-Ribot JL. Engineered control of cell morphology *in vivo* reveals distinct roles for yeast and filamentous forms of *Candida albicans* during infection. Eukaryot Cell. 2003;2(5):1053–60. 14555488 10.1128/EC.2.5.1053-1060.2003 PMC219382

[16] Dalle F, Wachtler B, L'Ollivier C, Holland G, Bannert N, Wilson D, et al Cellular interactions of *Candida albicans* with human oral epithelial cells and enterocytes. Cellular microbiology. 2010;12(2):248–71. Epub 2009/10/30. doi: CMI1394 [pii] 10.1111/j.1462-5822.2009.01394.x 19863559 10.1111/j.1462-5822.2009.01394.x

[17] Lorenz MC, Bender JA, Fink GR. Transcriptional response of *Candida albicans* upon internalization by macrophages. Eukaryot Cell. 2004;3(5):1076–87. 15470236 10.1128/EC.3.5.1076-1087.2004 PMC522606

[18] Kadosh D, Johnson AD. Induction of the *Candida albicans* filamentous growth program by relief of transcriptional repression: a genome-wide analysis. Molecular biology of the cell. 2005;16(6):2903–12. 15814840 10.1091/mbc.E05-01-0073 PMC1142434

[19] Nantel A, Dignard D, Bachewich C, Harcus D, Marcil A, Bouin AP, et al Transcription profiling of *Candida albicans* cells undergoing the yeast-to-hyphal transition. Molecular biology of the cell. 2002;13(10):3452–65. 12388749 10.1091/mbc.E02-05-0272 PMC129958

[20] Braun BR, Johnson AD. Control of filament formation in *Candida albicans* by the transcriptional repressor *TUP1* Science. 1997;277(5322):105–9. 9204892 10.1126/science.277.5322.105

[21] Braun BR, Kadosh D, Johnson AD. *NRG1*, a repressor of filamentous growth in *C*. *albicans*, is down-regulated during filament induction. EMBO J. 2001;20:4753–61. 11532939 10.1093/emboj/20.17.4753 PMC125265

[22] Murad AMA, Leng P, Straffon M, Wishart J, Macaskill S, MacCallum D, et al *NRG1* represses yeast-hypha morphogenesis and hypha-specific gene expression in *Candida albicans* . EMBO J. 2001;(20):4742–52. 11532938 10.1093/emboj/20.17.4742 PMC125592

[23] Zheng X, Wang Y, Wang Y. Hgc1, a novel hypha-specific G1 cyclin-related protein regulates *Candida albicans* hyphal morphogenesis. EMBO J. 2004;23(8):1845–56. 15071502 10.1038/sj.emboj.7600195 PMC394249

[24] Carlisle PL, Banerjee M, Lazzell A, Monteagudo C, Lopez-Ribot JL, Kadosh D. Expression levels of a filament-specific transcriptional regulator are sufficient to determine *Candida albicans* morphology and virulence. Proceedings of the National Academy of Sciences of the United States of America. 2009;106:599–604. 10.1073/pnas.0804061106 19116272 PMC2626749

[25] Mitchell AP. Dimorphism and virulence in *Candida albicans* . Current opinion in microbiology. 1998;1(6):687–92. 10066539 10.1016/s1369-5274(98)80116-1

[26] Brown AJ. Morphogenetic signaling pathways in *Candida albicans* In: Calderone RA, editor. *Candida* and Candidiasis. Washington, D.C.: ASM Press; 2002 p. 95–106.

[27] Brown AJ, Gow NA. Regulatory networks controlling *Candida albicans* morphogenesis. Trends Microbiol. 1999;7(8):333–8. 10431207 10.1016/s0966-842x(99)01556-5

[28] Biswas S, Van Dijck P, Datta A. Environmental sensing and signal transduction pathways regulating morphopathogenic determinants of *Candida albicans* . Microbiol Mol Biol Rev. 2007;71(2):348–76. Epub 2007/06/08. doi: 71/2/348 [pii] 10.1128/MMBR.00009-06 17554048 10.1128/MMBR.00009-06 PMC1899878

[29] Lu Y, Su C, Wang A, Liu H. Hyphal development in *Candida albicans* requires two temporally linked changes in promoter chromatin for initiation and maintenance. PLoS biology. 2011;9(7):e1001105 Epub 2011/08/04. doi: 10.1371/journal.pbio.1001105 PBIOLOGY-D-10-01188 [pii] 21811397 10.1371/journal.pbio.1001105 PMC3139633

[30] Banerjee M, Thompson DS, Lazzell A, Carlisle PL, Pierce C, Monteagudo C, et al *UME6*, a novel filament-specific regulator of *Candida albicans* hyphal extension and virulence Molecular biology of the cell. 2008;19(4):1354–65. 10.1091/mbc.E07-11-1110 18216277 PMC2291399

[31] Zeidler U, Lettner T, Lassnig C, Muller M, Lajko R, Hintner H, et al *UME6* is a crucial downstream target of other transcriptional regulators of true hyphal development in *Candida albicans* . FEMS Yeast Res. 2009;9(1):126–42. Epub 2008/12/05. doi: FYR459 [pii] 10.1111/j.1567-1364.2008.00459.x 19054126 10.1111/j.1567-1364.2008.00459.x

[32] Lu Y, Su C, Solis NV, Filler SG, Liu H. Synergistic Regulation of Hyphal Elongation by Hypoxia, CO2, and Nutrient Conditions Controls the Virulence of *Candida albicans* . Cell host & microbe. 2013;14(5):499–509. 10.1016/j.chom.2013.10.008 24237696 PMC4049569

[33] Guthrie C, Fink GR. Guide to yeast genetics and molecular biology. San Diego: Academic Press; 1991 xxxvii, 933 p.

[34] Liu H, Kohler J, Fink GR. Suppression of hyphal formation in *Candida albicans* by mutation of a *STE12* homolog. Science. 1994;266(5191):1723–6. 7992058 10.1126/science.7992058

[35] Lee KL, Buckley HR, Campbell CC. An amino acid liquid synthetic medium for the development of mycelial and yeast forms of *Candida albicans* . Sabouraudia. 1975;13(2):148–53. 808868 10.1080/00362177585190271

[36] Garcia-Sanchez S, Mavor AL, Russell CL, Argimon S, Dennison P, Enjalbert B, et al Global roles of Ssn6 in Tup1- and Nrg1-dependent gene regulation in the fungal pathogen, *Candida albicans* . Molecular biology of the cell. 2005;16(6):2913–25. 15814841 10.1091/mbc.E05-01-0071 PMC1142435

[37] Short JM, Fernandez JM, Sorge JA, Huse WD. Lambda ZAP: a bacteriophage lambda expression vector with *in vivo* excision properties. Nucleic acids research. 1988;16(15):7583–600. Epub 1988/08/11. 2970625 10.1093/nar/16.15.7583 PMC338428

[38] Childers DS, Mundodi V, Banerjee M, Kadosh D. A 5' UTR-mediated translational efficiency mechanism inhibits the *Candida albicans* morphological transition. Molecular microbiology. 2014;92(3):570–85. 10.1111/mmi.12576 24601998 PMC4032089

[39] Pfaffl MW. A new mathematical model for relative quantification in real-time RT-PCR. Nucleic acids research. 2001;29(9):e45 Epub 2001/05/09. 11328886 10.1093/nar/29.9.e45 PMC55695

[40] Lackey E, Vipulanandan G, Childers DS, Kadosh D. Comparative evolution of morphological regulatory functions in *Candida* species. Eukaryot Cell. 2013;12(10):1356–68. 10.1128/EC.00164-13 23913541 PMC3811340

[41] Schug J. Unit 2.6: Using TESS to Predict Transcription Factor Binding Sites in DNA Sequence Current Protocols in Bioinformatics: John Wiley and Sons; 2003. 10.1002/0471250953.bi0206s21 18428685

[42] Sellam A, Askew C, Epp E, Tebbji F, Mullick A, Whiteway M, et al Role of transcription factor CaNdt80p in cell separation, hyphal growth, and virulence in *Candida albicans* . Eukaryot Cell. 2010;9(4):634–44. 10.1128/EC.00325-09 20097739 PMC2863402

[43] Lassak T, Schneider E, Bussmann M, Kurtz D, Manak JR, Srikantha T, et al Target specificity of the *Candida albicans* Efg1 regulator. Molecular microbiology. 2011;82(3):602–18. 10.1111/j.1365-2958.2011.07837.x 21923768

[44] Lu Y, Su C, Liu H. A GATA transcription factor recruits Hda1 in response to reduced Tor1 signaling to establish a hyphal chromatin state in *Candida albicans* . PLoS pathogens. 2012;8(4):e1002663 Epub 2012/04/27. doi: 10.1371/journal.ppat.1002663 PPATHOGENS-D-11-02628 [pii] 22536157 10.1371/journal.ppat.1002663 PMC3334898

[45] Shapiro RS, Sellam A, Tebbji F, Whiteway M, Nantel A, Cowen LE. Pho85, Pcl1, and Hms1 signaling governs *Candida albicans* morphogenesis induced by high temperature or Hsp90 compromise. Curr Biol. 2012;22(6):461–70. Epub 2012/03/01. doi: S0960-9822(12)00123-6 [pii] 10.1016/j.cub.2012.01.062 22365851 10.1016/j.cub.2012.01.062

[46] Lu Y, Su C, Unoje O, Liu H. Quorum sensing controls hyphal initiation in *Candida albicans* through Ubr1-mediated protein degradation. Proceedings of the National Academy of Sciences of the United States of America. 2014;111(5):1975–80. 10.1073/pnas.1318690111 24449897 PMC3918812

[47] Enjalbert B, Whiteway M. Release from quorum-sensing molecules triggers hyphal formation during *Candida albicans* resumption of growth. Eukaryot Cell. 2005;4(7):1203–10. 10.1128/EC.4.7.1203-1210.2005 16002646 PMC1168956

[48] Lindsay AK, Deveau A, Piispanen AE, Hogan DA. Farnesol and cyclic AMP signaling effects on the hypha-to-yeast transition in *Candida albicans* . Eukaryot Cell. 2012;11(10):1219–25. 10.1128/EC.00144-12 22886999 PMC3485915

[49] Bockmuhl DP, Ernst JF. A potential phosphorylation site for an A-type kinase in the Efg1 regulator protein contributes to hyphal morphogenesis of *Candida albicans* . Genetics. 2001;157(4):1523–30. 11290709 10.1093/genetics/157.4.1523 PMC1461612

[50] Braun BR, Johnson AD. *TUP1*, *CPH1* and *EFG1* make independent contributions to filamentation in *Candida albicans* . Genetics. 2000;155(1):57–67. 10790384 10.1093/genetics/155.1.57 PMC1461068

[51] Tebarth B, Doedt T, Krishnamurthy S, Weide M, Monterola F, Dominguez A, et al Adaptation of the Efg1p morphogenetic pathway in *Candida albicans* by negative autoregulation and PKA-dependent repression of the *EFG1* gene. Journal of molecular biology. 2003;329(5):949–62. 12798685 10.1016/s0022-2836(03)00505-9

[52] Kaufmann E, Muller D, Knochel W. DNA recognition site analysis of *Xenopus* winged helix proteins. Journal of molecular biology. 1995;248(2):239–54. 7739038 10.1016/s0022-2836(95)80047-6

[53] Lotz H, Sohn K, Brunner H, Muhlschlegel FA, Rupp S. *RBR1*, a novel pH-regulated cell wall gene of *Candida albicans*, is repressed by *RIM101* and activated by *NRG1* . Eukaryot Cell. 2004;3(3):776–84. 15189998 10.1128/EC.3.3.776-784.2004 PMC420143

[54] Bensen ES, Martin SJ, Li M, Berman J, Davis DA. Transcriptional profiling in *Candida albicans* reveals new adaptive responses to extracellular pH and functions for Rim101p. Molecular microbiology. 2004;54(5):1335–51. 15554973 10.1111/j.1365-2958.2004.04350.x

[55] Kebaara BW, Langford ML, Navarathna DH, Dumitru R, Nickerson KW, Atkin AL. *Candida albicans* Tup1 is involved in farnesol-mediated inhibition of filamentous-growth induction. Eukaryot Cell. 2008;7(6):980–7. 10.1128/EC.00357-07 18424510 PMC2446655

[56] Cleary IA, Lazzell AL, Monteagudo C, Thomas DP, Saville SP. *BRG1* and *NRG1* form a novel feedback circuit regulating *Candida albicans* hypha formation and virulence. Molecular microbiology. 2012;85(3):557–73. Epub 2012/07/05. 10.1111/j.1365-2958.2012.08127.x 22757963 PMC3402693

