# Supplementary material for: Filament Condition-Specific Response Elements Control the Expression of NRG1 and UME6, Key Transcriptional Regulators of Morphology and Virulence in Candida albicans
Source: PLoS One. 2015 Mar 26;10(3):e0122775. doi: 10.1371/journal.pone.0122775 (PMC4374957; doi:10.1371/journal.pone.0122775)
Supplement: S1 Table — (PDF) [file pone.0122775.s001.pdf]

Table S1. **Strains used in this study**

Strain	Genotype	Source
SC5314	Prototroph	(1)
CAI4	*ura3Δ::imm*^{434}/*ura3Δ::imm*^{434} *iro1Δ::imm*^{434}/ *iro1Δ::imm*^{434}	(2)
DT425 (*UME6*$_{7\ kb\ URr}$-*lacZ*)	*ura3Δ::imm*^{434}/*ura3Δ::imm*^{434} *iro1Δ::imm*^{434}/*IRO1* *rps1::UME6*$_{7\ kb\ ur}$–*S.t. lacZ–URA3/RPS1*	This study
DT389 (*UME6*$_{6\ kb\ UR}$–*lacZ*)	*ura3Δ::imm*^{434}/*ura3Δ::imm*^{434} *iro1Δ::imm*^{434}/*IRO1* *rps1::UME6*$_{6\ kb\ ur}$–*S.t. lacZ–URA3/RPS1*	(3)
DT433 (*UME6*$_{5.5\ kb\ UR}$–*lacZ*)	*ura3Δ::imm*^{434}/*ura3Δ::imm*^{434} *iro1Δ::imm*^{434}/*IRO1* *rps1::UME6*$_{5.5\ kb\ ur}$–*S.t. lacZ– URA3/RPS1*	This study
DT437 (*UME6*$_{5\ kb\ UR}$–*lacZ*)	*ura3Δ::imm*^{434}/*ura3Δ::imm*^{434} *iro1Δ::imm*^{434}/*IRO1* *rps1::UME6*$_{5\ kb\ ur}$–*S.t. lacZ –URA3/RPS1*	This study
DT469 (*UME6*$_{4.9\ kb\ –\ 7\ kb\ UR}$–*lacZ*)	*ura3Δ::imm*^{434}/*ura3Δ::imm*^{434} *iro1Δ::imm*^{434}/*IRO1* *rps1::UME6*$_{4.9\ kb\ –\ 7\ kb\ ur}$–*S.t. lacZ–URA3/RPS1*	This study
DT472 (*UME6*$_{4.9\ kb\ –\ 6\ kb\ UR}$–*lacZ*)	*ura3Δ::imm*^{434}/*ura3Δ::imm*^{434} *iro1Δ::imm*^{434}/*IRO1* *rps1::UME6*$_{4.9\ kb\ –\ 6\ kb\ ur}$–*S.t. lacZ–URA3/RPS1*	This study
DT461 (*UME6*$_{4\ kb\ –\ 6\ kb\ UR}$–*lacZ*)	*ura3Δ::imm*^{434}/*ura3Δ::imm*^{434} *iro1Δ::imm*^{434}/*IRO1* *rps1::UME6*$_{4\ kb\ –\ 6\ kb\ ur}$–*S.t. lacZ–URA3/RPS1*	This study
DT457 (*UME6*$_{3\ kb\ –\ 6\ kb\ UR}$–*lacZ*)	*ura3Δ::imm*^{434}/*ura3Δ::imm*^{434} *iro1Δ::imm*^{434}/*IRO1* *rps1::UME6*$_{3\ kb\ –\ 6\ kb\ ur}$–*S.t. lacZ–URA3/RPS1*	This study
DT13 (*NRG1*$_{2.9\ kb\ UR}$–*lacZ*)	*ura3Δ::imm*^{434}/*ura3Δ::imm*^{434} *iro1Δ::imm*^{434}/*IRO1* *rps1::NRG1*$_{2.9\ kb\ ur}$–*S.t. lacZ–URA3/RPS1*	This study
DT1 (*NRG1*$_{2.1\ kb\ UR}$–*lacZ)*	*ura3Δ::imm*^{434}/*ura3Δ::imm*^{434} *iro1Δ::imm*^{434}/*IRO1* *rps1::NRG1*$_{2.1\ kb\ ur}$–*S.t. lacZ–URA3/RPS1*	This study

DT98 (NRG1$_{902\ bp\ UR}$–lacZ)	ura3Δ::imm^{434}/ura3Δ::imm^{434} iro1Δ::imm^{434}/IRO1 rps1::NRG1$_{902\ bp\ ur}$–S.t. lacZ–URA3/RPS1	This study
DT94 (NRG1$_{570\ bp\ UR}$–lacZ)	ura3Δ::imm^{434}/ura3Δ::imm^{434} iro1Δ::imm^{434}/IRO1 rps1::NRG1$_{570\ bp\ ur}$–S.t. lacZ–URA3/RPS1	This study
DT142 (NRG1$_{362\ bp\ UR}$–lacZ)	ura3Δ::imm^{434}/ura3Δ::imm^{434} iro1Δ::imm^{434}/IRO1 rps1::NRG1$_{362\ bp\ ur}$–S.t. lacZ–URA3/RPS1	This study
DT146 (NRG1$_{145\ bp\ UR}$–lacZ)	ura3Δ::imm^{434}/ura3Δ::imm^{434} iro1Δ::imm^{434}/IRO1 rps1::NRG1$_{145\ bp\ ur}$–S.t. lacZ–URA3/RPS1	This study
DT158 (NRG1$_{122\ bp\ UR}$–lacZ)	ura3Δ::imm^{434}/ura3Δ::imm^{434} iro1Δ::imm^{434}/IRO1 rps1::NRG1$_{122\ bp\ ur}$–S.t. lacZ–URA3/RPS1	This study
DT155 (NRG1$_{74\ bp\ UR}$–lacZ)	ura3Δ::imm^{434}/ura3Δ::imm^{434} iro1Δ::imm^{434}/IRO1 rps1::NRG1$_{74\ bp\ ur}$–S.t. lacZ–URA3/RPS1	This study
DT150 (NRG1$_{50\ bp\ UR}$–lacZ)	ura3Δ::imm^{434}/ura3Δ::imm^{434} iro1Δ::imm^{434}/IRO1 rps1:: NRG1$_{50\ bp\ ur}$–S.t. lacZ–URA3/RPS1	This study
DT370 (NRG1$_{45\ bp\ –\ 2.9\ kb\ UR}$–lacZ)	ura3Δ::imm^{434}/ura3Δ::imm^{434} iro1Δ::imm^{434}/IRO1 rps1::NRG1$_{45\ bp\ –\ 2.9\ kb\ \ bp\ ur}$–S.t. lacZ–URA3/RPS1	This study
DT374 (NRG1$_{342\ bp\ –\ 2.9\ kb\ UR}$–lacZ)	ura3Δ::imm^{434}/ura3Δ::imm^{434} iro1Δ::imm^{434}/IRO1 rps1::NRG1$_{342\ bp\ –\ 2.9\ kb\ ur}$–S.t. lacZ–URA3/RPS1	This study
DT362 (NRG1$_{556\ bp\ –\ 2.9\ kb\ UR}$–lacZ)	ura3Δ::imm^{434}/ura3Δ::imm^{434} iro1Δ::imm^{434}/IRO1 rps1::NRG1$_{556\ bp\ –\ 2.9\ kb\ ur}$–S.t. lacZ–URA3/RPS1	This study
DT366 (NRG1$_{883\ bp\ –\ 2.9\ kb\ UR}$ –lacZ)	ura3Δ::imm^{434}/ura3Δ::imm^{434} iro1Δ::imm^{434}/IRO1 rps1::NRG1$_{883\ bp\ –\ 2.9\ kb\ ur}$ –S.t. lacZ–URA3/RPS1	This study
DT378 (NRG1$_{1.9\ kb\ –\ 2.9\ kb\ UR}$–lacZ)	ura3Δ::imm^{434}/ura3Δ::imm^{434} iro1Δ::imm^{434}/IRO1 rps1::NRG1$_{1.9\ kb\ –\ 2.9\ kb\ ur}$ –S.t. lacZ–URA3/RPS1	This study
DT572 (NRG1$_{883\ bp\ –\ 1.5\ kb\ UR}$ –lacZ)	ura3Δ::imm^{434}/ura3Δ::imm^{434} iro1Δ::imm^{434}/IRO1 rps1::NRG1$_{883\ bp\ –\ 1.5\ kb\ ur}$ –S.t. lacZ–URA3/RPS1	This study
DT576 (NRG1$_{883\ bp\ –\ 2.1\ kb\ UR}$ –lacZ)	ura3Δ::imm^{434}/ura3Δ::imm^{434} iro1Δ::imm^{434}/IRO1 rps1::NRG1$_{883\ bp\ –\ 2.1\ kb\ ur}$ –S.t. lacZ–URA3/RPS1	This study

DT564 (*NRG1*$_{883\ bp\ –\ 2.5\ kb\ UR}$ *–lacZ*)	*ura3Δ::imm*^{434}*/ura3Δ::imm*^{434} *iro1Δ::imm*^{434}*/IRO1 rps1::NRG1*$_{883\ bp\ –\ 2.5\ kb\ ur}$ *–S.t. lacZ–URA3/RPS1*	This study
DT568 (*NRG1*$_{2.5\ kb\ –\ 2.9\ kb\ UR}$ *–lacZ*)	*ura3Δ::imm*^{434}*/ura3Δ::imm*^{434} *iro1Δ::imm*^{434}*/IRO1 rps1::NRG1*$_{2.5\ kb\ –\ 2.9\ kb\ ur}$*–S.t. lacZ–URA3/RPS1*	This study

UR = upstream region.

S.t. = *Streptococcus thermophilus*

References

1. Gillum AM, Tsay EY, Kirsch DR (1984) Isolation of the *Candida albicans* gene for orotidine-5'-phosphate decarboxylase by complementation of *S. cerevisiae ura3* and *E. coli pyrF* mutations. Mol Gen Genet 198:179-82.
2. Fonzi WA, Irwin MY (1993) Isogenic strain construction and gene mapping in *Candida albicans*. Genetics 134:717-28.
3. Childers DS, Mundodi V, Banerjee M, Kadosh D (2014) A 5’ UTR-mediated translational efficiency mechanism inhibits the *Candida albicans* morphological transition. Mol Microbiol 92:570-85.
