# Supplementary material for: Filament Condition-Specific Response Elements Control the Expression of NRG1 and UME6, Key Transcriptional Regulators of Morphology and Virulence in Candida albicans
Source: PLoS One. 2015 Mar 26;10(3):e0122775. doi: 10.1371/journal.pone.0122775 (PMC4374957; doi:10.1371/journal.pone.0122775)
Supplement: S2 Table — (PDF) [file pone.0122775.s002.pdf]

Table S2 **Primers used in this study**

#	Primer Name	Sequence	Description
1	DTO13	catgtcaagtctcgagGCATAATGATTGTTTGATTCTTAATGAAAC	3’ *NRG1* 2.9 kb promoter primer for cloning into placbasal
2	DTO33	catgtgcaattgtactgcagCTTGCAACATTGTGTAGTTGC	5’ *NRG1* 2.9 kb promoter primer for cloning into placbasal
3	DTO228	agtactgaactgcagCAAAACCCCAATTGCTTGGC	5’ *NRG1* 2.5 kb promoter primer for cloning into placbasal
4	DTO30	catgtgcaattgtactgcagCACAATAGCATACTATGCAGGC	5’ *NRG1* 2.1 kb promoter primer for cloning into placbasal
5	DTO229	agtactgaactgcagGAGATGCACTTGGTGGTATACC	5’ *NRG1* 1.5 kb promoter primer for cloning into placbasal
6	DTO83	agtactgaactgcagGCACATAGCCATGCATTAGCC	5’ *NRG1* 902 bp promoter primer for cloning into placbasal
7	DTO84	agtactgaactgcagGCTAGCAATGAACCAAACGG	5’ *NRG1* 570 bp promoter primer for cloning into placbasal
8	DTO154	agtactgaactgcagCGTCCAGGTTACTATTTTCC	5’ *NRG1* 362 bp promoter primer for cloning into placbasal
9	DTO182	agtactgaactgcagCATTGTTCAAGTATCTTCC	5’ *NRG1* 122 bp promoter primer for cloning into placbasal
10	DTO181	agtactgaactgcagCAATAAAACATCGTTATCC	5’ *NRG1* 74 bp promoter primer for cloning into placbasal
11	DTO156	agtactgaactgcagCATCTCAAAATTTTTCCCTGC	5’ *NRG1* 50 bp promoter primer for cloning into placbasal
12	DTO230	agtactgaactcgagGATGAGAAACAGGATAACG	3’ *NRG1* 45 bp promoter primer for cloning into

			placbasal
13	DTO237	agtactgaactcgagGGAAAATAGTAACCTGGACG	3' *NRG1* 342 bp promoter primer for cloning into placbasal
14	DTO233	agtactgaactcgagGGTTCATTGCTAGCTTGTTAGG	3' *NRG1* 556 bp promoter primer for cloning into placbasal
15	DTO234	agtactgaactcgagCTAATGCATGGCTATGTGCC	3' *NRG1* 883 bp promoter primer for cloning into placbasal
16	DTO235	agtactgaactcgagGACCACCTCAAGCCTTGG	3' *NRG1* 1.9 kb promoter primer for cloning into placbasal
17	DTO250*	agtactgaactgcaggcatgcTTATACAGTGGATTAAAAAATAAAACTAATTGGAAGTAAATTGAGG	3' *UME6* promoter downstream primer for cloning into placbasal
18	DTO90*	CTGTTAAGTTGTTGTGGTAGG	3' *UME6* promoter primer downstream of native HindIII site for cloning into placbasal
19	DTO89*	GGTCCAGTCGACCAAGAACAATCCATTCTCG	5' *UME6* promoter primer upstream of native HindIII site for cloning into placbasal
20	DTO243*	agtactgaactgcaggcatgcTTATACAGTGGATTAAAAAATAAAACTAATTGGAAGTAAATTGAGG	5' 6.0 kb *UME6* promoter upstream primer for cloning into placbasal
21	DTO255	agtactgaactgcagGAGGCAAATGGCAAGCACC	5' 7.0 kb *UME6* promoter upstream primer for cloning into placbasal
22	DTO110	CAACATTATCACCTAGACCC	3' *UME6* promoter primer downstream of native XhoI site for cloning into placbasal
23	DTO123	GGTTACCAAAACATATCTCG	5' *UME6* promoter primer upstream of native XhoI site for cloning into placbasal
24	DTO254	agtactgaactgcagGTTGAAATGCACATATCGG	5' 5.5 kb *UME6* promoter upstream primer for

			cloning into placbasal
25	DTO93	GCAACTCCCGGGAAATTCTA	3’ *UME6* promoter primer downstream of native HindIII and SmaI sites for cloning into placbasal
26	DTO51	caatcgattctgcagGATATGTCTTTGTGTTGC	5’ 5.0 kb *UME6* promoter upstream primer for cloning into placbasal
27	DTO252	agtactgaactgcaggcatgcCCTTACTTTTCTAGTTCC	3’ 3.0 kb *UME6* promoter downstream primer for cloning into placbasal
28	DTO251	agtactgaactgcaggcatgcGAATGAAGTTGGAGTGGC	3’ 4.0 kb *UME6* promoter downstream primer for cloning into placbasal
29	DTO260	agtactgaagcatgcCATGCAGCAAGTAATTTGC	3’ 4.9 kb *UME6* promoter downstream primer for cloning into placbasal
30	DTO169*	CGTCTTTGCAGATCGTACCC	5’ *lacZ* primer for qRT-PCR analysis
31	DTO170*	CTGGGAATGTTGCTTCTTCG	3’ *lacZ* primer for RT-qPCR analysis
32	DTO85*	TTGCTCCAGAAGAACATCCAG	5’ *ACT1* primer for RT-qPCR analysis
33	DTO86*	AGTAACACCATCACCAGAATCC	3’ *ACT1* primer for RT-qPCR analysis
34	DTO155	agtactgaactgcagCAATTTGCTTTACAAGAGAACC	5’ *NRG1* 145 bp promoter primer for cloning into placbasal
35	DTO270	agtactgaactcgagGTGTTTGTCCTAGATTGCG	3’ *NRG1* 2.5 kb promoter primer for cloning into placbasal

Note: Lowercase bases indicate flanking sequences within primers that contain restriction sites for cloning.

*previously described in Childers DS, Mundodi V, Banerjee M, Kadosh D (2014) A 5’ UTR-mediated translational efficiency mechanism inhibits the *Candida albicans* morphological transition. Mol Microbiol 92:570-85.
